# Cleaner Biofuel Production via Process Parametric Optimization of Nonedible Feedstock in a Membrane Reactor Using a Titania-Based Heterogeneous Nanocatalyst: An Aid to Sustainable Energy Development

**DOI:** 10.3390/membranes13120889

**Published:** 2023-11-27

**Authors:** Maria Ameen, Muhammad Zafar, Mushtaq Ahmad, Mamoona Munir, Islem Abid, Abd El-Zaher M. A. Mustafa, Mohammad Athar, Trobjon Makhkamov, Oybek Mamarakhimov, Akramjon Yuldashev, Khislat Khaydarov, Afat O. Mammadova, Laziza Botirova, Zokirjon Makkamov

**Affiliations:** 1 Department of Plant Sciences, Quaid-i-Azam University Islamabad, Capital Territory, Islamabad 15320, Pakistanmushtaqflora@hotmail.com (M.A.); 2 Pakistan Academy of Sciences, Constitution Avenue, G-5/2 G-5, Islamabad 44000, Pakistan; 3 Department of Botany, Rawalpindi Women University, 6th Rd., Satellite Town, Rawalpindi Punjab 46300, Pakistan; 4 Department of Botany and Microbiology, College of Science, King Saud University, P.O. Box 2455, Riyadh 11451, Saudi Arabia; iabid@ksu.edu.sa (I.A.); amus@ksu.edu.sa (A.E.-Z.M.A.M.); 5 California Department of Food and Agriculture, Pest Detection & Emergency Projects, 1220 ‘N’ Street, 2nd Floor, Sacramento, CA 95814, USA; athar.tariq@cdfa.ca.gov; 6 Department of Forestry and Landscape Design, Tashkent State Agrarian University, 2 A., Universitet Str., Kibray District, Tashkent 100700, Uzbekistan; 7 Department of Ecological Monitoring, National University of Uzbekistan, 4 University Street, Tashkent 100174, Uzbekistan; 8 Department of Ecology and Botany, Andijan State University, 129, Universitet Str., Andijan 170100, Uzbekistan; 9 Institute of Biochemistry, Samarkand State University, University blv. 15, Samarkand 140104, Uzbekistan; 10 Department of Botany and Plant Physiology, Baku State University, Baku 1148, Azerbaijan; 11 Department of Medicinal Plants and Botany, Gulistan State University, 4, Micro-District, Gulistan, Sirdarya 120100, Uzbekistan; 12 Department of Customs Regulation and Customs Payments, Customs Institute of the Customs Committee of the Republic of Uzbekistan, Qazirabad 2-Street, 118, Tashkent 100071, Uzbekistan

**Keywords:** biodiesel, membrane reactor, response surface methodology, Titania nanoparticles

## Abstract

Membrane technology has been embraced as a feasible and suitable substitute for conventional time- and energy-intensive biodiesel synthesis processes. It is ecofriendly, easier to run and regulate, and requires less energy than conventional approaches, with excellent stability. Therefore, the present study involved the synthesis and application of a highly reactive and recyclable Titania-based heterogeneous nanocatalyst (TiO_2_) for biodiesel production from nonedible *Azadhiracta indica* seed oil via a membrane reactor, since *Azadhiracta indica* is easily and widely accessible and has a rich oil content (39% *w*/*w*). The high free fatty acids content (6.52 mg/g KOH) of the nonedible oil was decreased to less than 1% via two-step esterification. Following the esterification, transesterification was performed using a heterogeneous TiO_2_ nanocatalyst under optimum conditions, such as a 9:1 methanol–oil molar ratio, 90 °C reaction temperature, 2 wt.% catalyst loading, and an agitation rate of 600 rpm, and the biodiesel yield was optimized through response surface methodology (RSM). *Azadhiracta indica* seed oil contains 68.98% unsaturated (61.01% oleic acid, 8.97% linoleic acid) and 31.02% saturated fatty acids (15.91% palmitic acid, 15.11% stearic acid). These fatty acids transformed into respective methyl esters, with a total yield up to 95% achieved. The biodiesel was analyzed via advanced characterization techniques like gas chromatography–mass spectrometry (GC-MS), Fourier transform infrared spectroscopy (FT-IR), and nuclear magnetic resonance (NMR), whereas the catalyst was characterized via X-ray diffraction (XRD), scanning electron microscopy (SEM), energy-dispersive X-ray (EDX), and Fourier transform infrared spectroscopy (FT-IR). Due to its physicochemical properties, *Azadirachta indica* seed oil is a highly recommended feedstock for biodiesel production. Moreover, it is concluded that the Titania-based heterogeneous nanocatalyst (TiO_2_) is effective for high-quality liquid fuel synthesis from nonedible *Azadirachta indica* seed oil in a membrane reactor, which could be an optional green route to cleaner production of bioenergy, eventually leading to sustenance, robustness, and resilience that will aid in developing a holistic framework for integrated waste management.

## 1. Introduction

The rapid depletion of oil reserves, petroleum price hikes, and the adversarial effects of fossil fuel resources on the environment are the key factors that lead to the pursuit of a replacement energy source. Biofuels, particularly biodiesel, are an attractive renewable source of energy that is produced from biomass using a different extraction or conversion process [1,2]. Biodiesel, owing to its nontoxic nature, with lower levels of carbon monoxide, sulfur dioxide, and particulate matter pollution, presents a viable substitute for petroleum diesel [3]. Biodiesel production utilizes a variety of feedstock such as vegetable oil, animal fat, waste cooking oil, algal oil, grease, etc. Researchers have developed various procedures in search of a more sustainable and effective method for biodiesel production [4]. Conventional methods involve the reaction between triglycerides in oil or fat and methanol in the presence of a homogeneous base catalyst, primarily NaOH or KOH. The drawbacks of using homogeneous catalysts include saponification and challenges in their recovery. Furthermore, water is used in this method for biodiesel purification, which results in an increased production cost. The economic viability and profitability of biodiesel production through homogeneous catalysts are especially lower when compared with the total expenses of biodiesel production from fossil fuels [5]. Therefore, it becomes essential to explore an alternative way that offers clean burning, increased efficiency, and environmentally friendly operations with reduced corrosive properties. This approach should also ensure the easy recovery of the catalyst from the end product, i.e., biodiesel [6,7].

**Figure 1:**
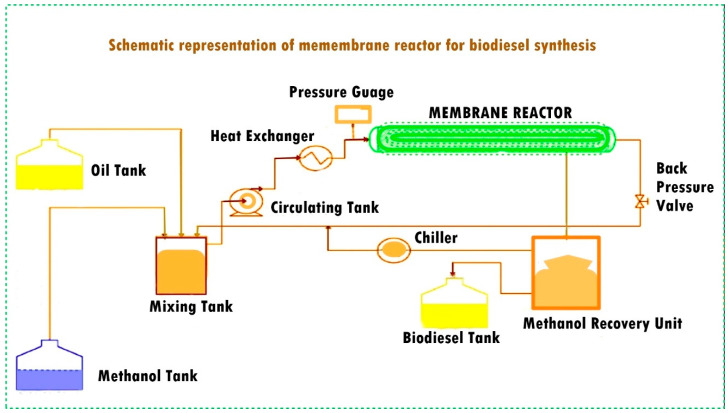
Biodiesel synthesis process via membrane reactor [20].

In this context, the heterogeneous catalysis facilitated by a membrane reactor emerges as a viable solution to address the challenges posed by the homogeneous process. Heterogeneous catalysts encompass acids, bases, bifunctional catalysts, ion exchange resins, hydrotalcites, and biocatalysts. Specifically, heterogeneous catalysts include metal oxides of alkali metals, alkaline earth metals, and transition metals. Some examples of these metal oxides are CaO, MgO, CdO, Ag_2_O, ZnO, BaO, CoO, ZrO, CuO, and NiO, which have been utilized as a catalyst in the biodiesel synthesis process with high conversion yields [8]. Among these metal oxides, titanium dioxide (TiO_2_) stands out as a versatile and cost-effective catalyst with various applications, including petrochemical and petroleum refining [9]. Sulfated TiO_2_, in particular, serves as a solid superacidic catalyst with enhanced performance due to its high acid strength and Brönsted acidity, reducing the catalytic deactivation. [Ti(SO_4_)O], a novel solid acid nanocatalyst developed by Hassanpour et al. for petrochemical conversion and petroleum refining, was shown to be able to achieve a 97.1% yield of fatty acid methyl esters (FAMEs) from used cooking oil (UCO) under a reaction time of 3 h, a catalyst–UCO ratio of 1.5 wt.%, and a 9:1 methanol–UCO ratio at a 75 °C reaction temperature, and the biodiesel satisfied the ASTM D6751 and EN 14214 standards [10]. Oprescu et al. reported another source for biodiesel production, i.e., microalgae oil feedstock and an amphiphilic SO_4_^2−^/TiO_2_-ZrO_2_ superacid catalyst along with the transesterification over KOH. Wang et al. used SO_4_^2−^/TiO_2_-SiO_2_ as a solid acid catalyst for the simultaneous esterification and transesterification of low-cost feedstocks with a high FFA content such as the waste cooking oil (WCO) catalyzed using a solid acid catalyst SO4^2−^/TiO_2_/La^3+^ and up to 96% yield was achieved [11]. Viswanathan et al. synthesized sulfated Fe_2_O_3_/TiO_2_ (SFT) through calcination at different temperatures (300–900 °C) with over 90% conversion [12]. Afolabi et al. investigated the catalytic characteristics of a mixed metal oxide TiO_2_-supported ZnO catalyst for the conversion of waste cooking oil into biodiesel in the presence of methanol and hexane as cosolvent (1:1); this approach led to a 91.3% yield [13]. Wen et al. converted waste cooking oil into biodiesel using mixed oxides of MgOTiO_2_ (MT) synthesized via the sol gel technique to achieve a 92.3% yield [14]. Kawashima et al. [15] performed transesterification of rapeseed oil using heterogeneous base catalyst; a 90% methyl ester yield was obtained. Baroutian et al. [16] used a TiO_2_/Al_2_O_3_ membrane reactor for biodiesel production and investigated the effect of reaction temperature, catalyst concentration, and cross-flow circulation velocity. Biodiesel was produced from palm oil using alkali catalyst in a ceramic membrane reactor with 1.12 wt.% catalyst concentration, at 70 °C reaction temperature and a cross-flow circulation velocity of 0.211 cms^−1^; a biodiesel yield of 95.1% was achieved. Biodiesel production in a membrane reactor has several advantages. Membrane reactors provide higher reaction rates with faster conversion of feedstock into the biodiesel through the optimization of the reaction conditions. This alternative method allows easy catalyst recovery and recycling. The controlled environment of a membrane reactor promotes the selective process, reduces the formation of undesirable byproducts, enables simultaneous reaction and separation thus facilitates the continuous removal of biodiesel from the reaction mixture. It prevents reverse reactions, thus improving the purity of the biodiesel. Moreover, the product separation that occurs during the process shifts the equilibrium forward, i.e., towards the biodiesel synthesis [17]. Membrane reactors have a smaller unit as compared to the conventional reactors and separation units; making them suitable for small-scale biodiesel production. They contribute to process intensification through improving the reaction kinetics and reducing the mass transfer limitations for higher production rates within the same system. Membrane reactors can process a wide range of feedstocks, including low-quality oils with high free fatty acid content for biodiesel production [18]. Higher conversion rates with low waste makes the biodiesel synthesis process more ecofriendly. The use of membrane reactors for biodiesel synthesis drives ongoing research and innovation, resulting in the development of more efficient catalysts, membranes, and process designs [19]. Figure 1 shows the schematic representation of biodiesel synthesis in membrane reactor.

Figure 1 elaborates that the feedstock is fed into the membrane reactor, where the reaction and separation occur simultaneously. This integration reduces the need for additional washing and recycling limitations because of the improved contact between the reactants inside the membrane–catalyst interface, leading to high conversion, p [21]. The application of enhanced membrane technologies in wastewater treatment and filtration processes has been extensively investigated in a number of research projects. This study is explained by these technologies’ capacity to separate different components inside a single process unit, especially in relation to molecular size [20]. This method eliminates the need for water during the whole process by combining the simultaneous reaction and component separation into a single process stream. Membrane reactors demonstrate their ability to achieve selectivity by allowing only molecules with smaller molecular sizes to pass through while keeping molecules with larger molecular sizes intact [22]. Additionally, this process improves the interaction between the solid catalyst and the insoluble feedstock (oil), which yields the best possible production yield [23,24]. Because water resources can be scarce, synthesizing biodiesel using membrane technologies has the added benefit of saving water, as it does not require further purification or wastewater treatment. Additionally, this shift improves the environmental friendliness of the approach by reducing pollution [25]. An earlier investigation used palm oil as the feedstock and KOH as the reaction catalyst, but it was unable to sufficiently address the problems of selectivity and the presence of undesirable products like glycerin in the final product stream [26].

Moreover, the refinement procedure plays a central role in improving the efficacy of biodiesel production. Generally, biodiesel production methods involve the optimization of specific reaction variables, which results in time and cost ineffectiveness. This method has passed over the intricate interactions between independent parameters and their complete influence on the production process. On the other hand, the application of response surface methodology (RSM) offers a multidimensional view by analyzing the interaction of reaction variables through statistical techniques. The experimental design using RSM can accurately predict the reactions under various transesterification conditions, making it highly valuable for large-scale biodiesel production.

In the current study, *Azadirachta indica* (Neem) was selected as the feedstock for biodiesel production. *Azadirachta indica* yields about 40–50 kg of fruits per plant every year. It has a 2.67 ton/ha average seed output at 400 plants per hectare. The seed kernel capacity to produce neem oil ranges from 35 to 45%. The implementation of biofuels as a substitute for conventional fuels has led to a notable transformation in the technological approaches to energy assessment. Specifically, vegetable oils such as *Azadirachta indica* oil have undergone successful conversions into biodiesel, triggering a significant change in the characteristics of the fuel produced [27]. Physicochemical analyses of biodiesel reveal striking resemblance to regular petrodiesel. However, the primary challenge faced in the production of *Azadirachta indica* biodiesel (AIBD) is the struggle to lower the production costs to a level at which it can competitively coexist with conventional diesel fuel [24,25,28].

To the best of our knowledge, there has been relatively little research on the catalytic function of TiO_2_ nanoparticles in biodiesel production from *Azadirachta indica* seed oil in a membrane reactor. The main objective of this study was to prepare a high-quality biodiesel in a membrane reactor using a Titania-based nanocatalyst. The novel contribution of this study lies in its innovative membrane reactor engineering design. This design incorporates a micro-filtrated membrane with an exceptionally small pore size, supported by a Titania nanocatalyst to enhance the surface reaction interfaces. This configuration ensures an effective retention of free glycerol and unreacted oil in the reaction medium, where these components undergo decomposition and molecular restructuring into biodiesel and methanol at the membrane filtration junction. The impregnation of Titania nanocatalysts into the membrane matrix not only increases the reactive surface but also narrows the filtration membrane pores, resulting in a synergistic mechanism for high-quality biodiesel production.

## 2. Materials and Methods

### 2.1. Oil Determination and Extraction

Solvent extraction or leaching is a process by which a chemical constituent is removed from a sample by means of an organic solvent [29]. In this study, Soxhlet apparatus was used for the detection of the oil content in *Azadirachta indica* seeds. Seeds were oven-dried at 55 °C for one night and crushed into a fine powder (5 g). Then, 250 mL of n-Hexane was poured into a round-bottomed flask in the Soxhlet apparatus. A thimble containing seed powder (5 g) was inserted in the center of the apparatus. As the solvent in the round-bottomed flask heated up to 60 °C, it boiled and vaporized through the condenser fitted at the top; then, the condensed solvent dripped into the thimble which held the powdered sample to be extracted. The extract leached down through the pores of the thimble and filled the siphon tube, where it flowed down into the round-bottomed flask. Oil droplets were clearly visible in the round-bottomed flask and the solvent was repeatedly recovered and reused. This process was allowed to continue for 6 h. The resulting wet sample in the thimble was kept in the oven. When the solvent evaporated and the sample had completely dried, it was weighed. The weight of the sample was determined to enable us to calculate the amount of oil that had been extracted. The reduction in the weight of the sample was attributed to the extraction of the oil from it [30,31].

For mechanical extraction, an electric oil expeller (KEK P0015, 10127, Remscheid, Germany) was used to expel oil from the *Azadirachta indica* seeds (10 kg) and the crude oil was collected in bottles for further processing. After 3–5 turns, the oil was completely expelled, followed by filtration using Whattman filter paper No. 42 (Shanghai, China).

### 2.2. Free Fatty Acid Content

An aqueous acid-based titration process was performed to determine the FFA content of the *Azadirachta indica* seed oil. For blank titration, potassium hydroxide (0.025 M) was slowly poured into 10 mL of isopropanol in the presence of phenolphthalein as an indicator. Then, a sample titration process was carried out using a known quantity of seed oil. FFA was calculated using Equation (1) [32]:

(1) $$\mathrm{FFA}\;\mathrm{content}=\frac{(A-B)\times C}{V}$$

A = volume used in sample titration; B = volume of KOH used in blank titration; C = conc. of KOH; V = weight of oil used (g) in sample titration.

### 2.3. Catalyst Synthesis

The catalyst synthesis involved titanium isopropoxide (C_12_H_28_O_4_Ti) and isopropanol. Through uniform agitation, titanium isopropoxide (5mL) was gradually added to the isopropanol (15 mL) at 40 °C and stirred for 20 min. Then, polyvinylpyrroliperformed (0.1 g) was added along with the distilled water (10 cc). Nitric acid (HNO_3_) was used to maintain its pH, which resulted in the appearance of white precipitates of Ti(OH)_4_. Ti(OH)_4_ was separated through spinning, and different contaminants were continuously eliminated through soaking the Ti(OH)_4_ in purified water. The purified precipitates were allowed to dry in an oven at 80 °C for 24 h. The desiccated and a finely ground Ti(OH)_4_ transformed into titanium dioxide upon calcination at 570 °C for 3 h. Ti(OH)_4_ underwent a significant transition into titanium dioxide at temperatures above 400 °C [33].

### 2.4. Catalyst Characterization

X-ray diffraction (XRD) was used to identify the crystallographic arrangement of the titanium dioxide nanocatalyst. XRD analysis was performed on a copper-equipped diffractometer (40 kV) using a current voltage of 35 mA. The sample was analyzed over a 2θ scale range of 30–80° with scanning rate of 0.5°/min [34].

The comprehensive morphological structure of the nanocatalyst was identified using scanning electron microscopy (SEM). For SEM analysis, a drop of TiO_2_ nanocatalyst suspension was poured on an SEM grid. SEM was performed following gold sputtering (JOEL-JSM-5910, Peabody, MA, USA). An energy-dispersive X-ray spectrum in the range of 0–1350 eV was used to investigate the sample composition and to perform an elemental analysis of the TiO_2_ nanocatalyst [35,36].

Fourier transform infrared spectroscopy (FT-IR) was performed using a Bruker-Tensor (Billerica, MA, USA) 27 in a range of 400–4000 cm^−1^. The resolutions of the scans for analyses were 1 cm^−1^ and 15 cm^−1^; these were used to examine the functional groups in the *Azadirachta indica* methyl esters [37].

### 2.5. Acid Esterification

*Azadirachta indica* seed oil was subjected to acid esterification to lower the high FFA content. The pretreatment was performed at 50 °C for 1 h with an acid catalyst (1% *w*/*w* H_2_SO_4_) and the mixture was left to settle for 2 h, resulting in the formation of two layers which were separated from each other; a layer of oil was processed for the next step, i.e., transesterification [5].

### 2.6. Membrane Reactor Design

A membrane reactor with a titanium dioxide/aluminum oxide membrane, with the tubular element being enclosed in PVC (Atech Innovations, Gladbeck, Germany), was used to perform an effective conversion of *Azadhiracta indica* seed oil into biodiesel. To ensure that the membrane had an entrance and an exit, sieve plates (154 µm) were integrated into the membrane itself. The specific dimensions of the reactor were 42 cm length, 1.7 cm internal diameter, 2.55 cm exterior diameter, and 0.0211 m^2^ of the filtration surface area. A pore size of approximately 0.043 µm was adopted for the retention of the oil molecules within the membrane to achieve a high biodiesel yield. The raw material was then injected into the membrane reactor where the reaction and separation simultaneously occur as an integrated process, eliminating the need for additional washing or recycling constraints. This results in a higher conversion of the feedstock into biodiesel due to increased contact among the reactants in the membrane–catalyst interphase.

### 2.7. Transesterification

*Azadhiracta indica* seed oil and a mixture of methanol and the Titania-based heterogeneous catalyst were charged into the system using two raw material pumps. A third pump (circulating pump) was used to continually charge the reactor with the methanol/TiO_2_ mixture. Then, it was ‘turned on’ to heat up the reactant; meanwhile, *Azadhiracta indica* seed oil was added to the reactor. Two pressure gauges were used to maintain the pressure inside the membrane. In a round-bottomed flask (a part of the methanol-recovery unit), a permeating stream containing biodiesel, glycerol, methanol, and titanium dioxide was collected. To reduce consumption, the methanol—one of the transesterification reactants with a lower boiling point—was evaporated, distilled, and then added back into the system. After an hour, the circulating pump and heat exchanger were switched off and the products were transferred into a separating funnel to separate the biodiesel from the glycerol byproduct [38].

Methanol molecules, due to their small molecular size, are able to pass through the membrane along with biodiesel and glycerol. Methanol must be recycled and added back into the transesterification process because it is a crucial reactant. The overall methanol–oil ratio is reduced through recycling the methanol from the permeating stream. To reduce consumption, methanol, which has a lower boiling point, was continuously evaporated, distilled, and reintroduced into the system. A Leibig condenser, a three-neck round-bottomed flask, an oil bath, and a thermometer are the basic components of a distillation unit that is called a methanol-recovery unit. In the three-neck round-bottomed flask, the permeating stream, containing biodiesel, methanol, and glycerol, was collected. The high temperature of the oil bath leads to immediate methanol evaporation. The excess methanol was removed from the biodiesel layer through evaporation.

**Figure 2:**
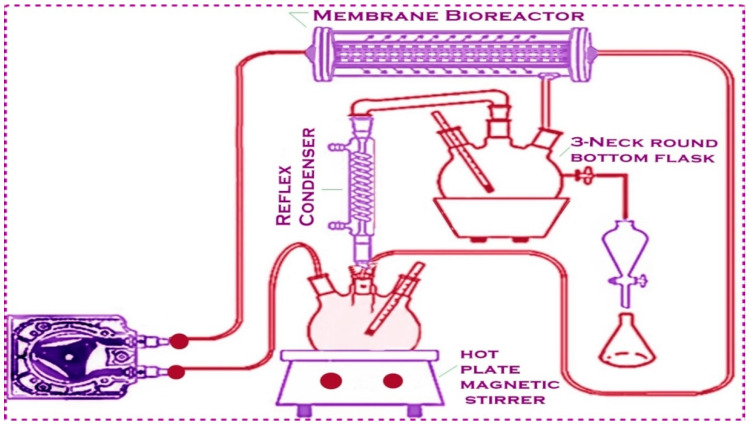
Transesterification process in membrane reactor.

The pure biodiesel was further analyzed using GC-MS, FT-IR, and NMR. The system was flushed with pure ethanol for 30 min and then drained after each run. Because of the excellent chemical and thermal stability of the membrane, no real evidence of change in its performance was observed after almost one year of operation and contact with the methanol and base solution [38,39,40]. Figure 2 shows a membrane reactor designed for biodiesel production.

The optimization of the biodiesel synthesis process using *Azadhiracta indica* seed oil as feedstock within a fixed-bed membrane reactor was conducted in alignment with the established framework and input criteria.

Biodiesel yield (%) = Biodiesel-produced (g) ÷ Used-Oil (g) × 100(2)

### 2.8. Optimization Study and Design of Experiment

Using design expert-13 from stat-ease Inc., Minneapolis, Minnesota, a response surface methodology (RSM) based on the box Behnken design (BBD) was developed to examine the effects of the various independent variables, such as the methanol–oil molar ratio (Met/Oil), the catalyst concentration, the reaction time, and the reaction temperature. Four parameters with maximum and minimum value ranges endured thirty trials with a Met/Oil of 3:1–15:1, a reaction time of 60–360 min, a catalyst concentration of 0.5–3.5 wt.%, and a reaction temperature of 60–120 °C.

### 2.9. Biodiesel Characterization

By means of a 300 MHz spectrometer (Avan CE) with BBO (5 mm) probes (7.05 T), deuterated chloroform CDCl_3_ was used as a solvent and tetramethyl silane TMS was used as an internal standard; the process was performed at 25 °C, with a 300-pulse duration, 8 scans, and a second recycle delay. Accordingly, the ^1^HNMR and ^13^CNMR spectra of the *Azadhiracta indica* biodiesel were obtained [41]. The percentage conversion of triglycerides into methyl esters was found using the following Equation (3):

C = 100 × 2AMe/3ACH_2_ (3)

C = oil conversion into methyl esters; AMe = integration position of −OCH_3_; ACH_2_ = position of integration of α – CH_2_.

The FT-IR spectrometer (bruker-tensor 27) was used to obtain the infrared spectrum, using a scan rate of 15 at 1 cm^−1^ and an effective wavelength of 4000–400 cm^−1^ [42].

Gas chromatography–mass spectrometry (GC-MS) (DB-5MS- Agilent, 0.25 mm column, 30–0.32 mm) was used to detect the existence of various methyl esters [43,44]. Additionally, helium was used as a carrier gas for the 120–300 °C automatic column temperature (10 °C/min). However, the fuel properties were compared according to the international American Standard for Testing Material (ASTM).

## 3. Results

### 3.1. Catalyst Characterization

**Figure 3:**
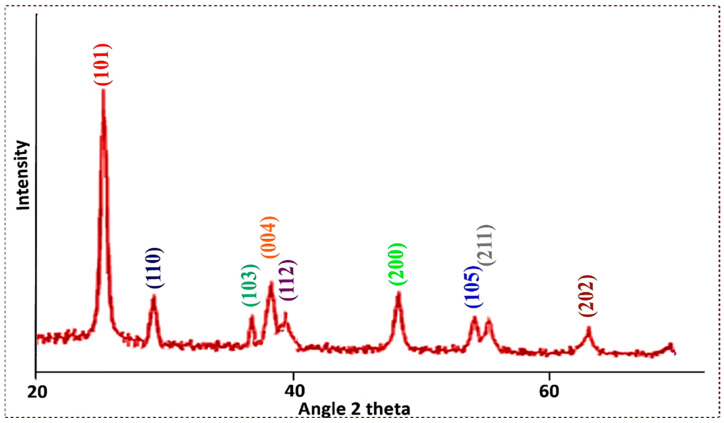
XRD graph of Titania-based nanocatalyst.

The titanium dioxide nanopowder (TiO_2_) was characterized using X-ray diffraction analysis to determine its crystalline structure. The XRD pattern showed a rutile configuration and an anatase phase of TiO_2_ with strong diffraction peaks at 2θ angles, i.e., 25.313° (101), 26.49° (110), 37.10° (103), 37.83° (004), 38.63° (112), 48.089° (200), 53.99° (105), 55° (211), and 63.5° (202). The intensity of the peak is high and the width of the (101) plane at 2θ = 25.313° becomes narrow (Figure 3). The sharpness of the peak shows that the samples possess a good crystalline nature. The strong diffraction peaks around 25.313° and 48.089° indicate that the TiO_2_ nanoparticles have tetragonal symmetry in the anatase phase.

The average particle size was calculated using the Debye Scherer equation:

(4) $$D=\frac{K\times \lambda \;(nm)}{\beta cos\theta }$$

where D = crystalline size in nanometer; K = Scherer constant (0.98); λ = wavelength in nanometer (1.54); θ = Braggs angle, β = full width at half maximum (FWHM).

The average particle size was found to be 42 nm and K is an integer which is given as 0.89. λ is the wavelength of the X-ray radiation (CuK = 0.15 nm). Farbod and Khademalrasool [45] and Salari and Marashi [46] reported average crystalline sizes for TiO_2_ of 37 nm and 15 nm, respectively, by adopting mechano–chemical syntheses. XRD data of titanium dioxide nanoparticles have been reported in previous publications with consistent findings; these nanoparticles have been successfully used to produce biodiesel from a variety of nonedible feedstocks [47,48,49].

**Figure 4:**
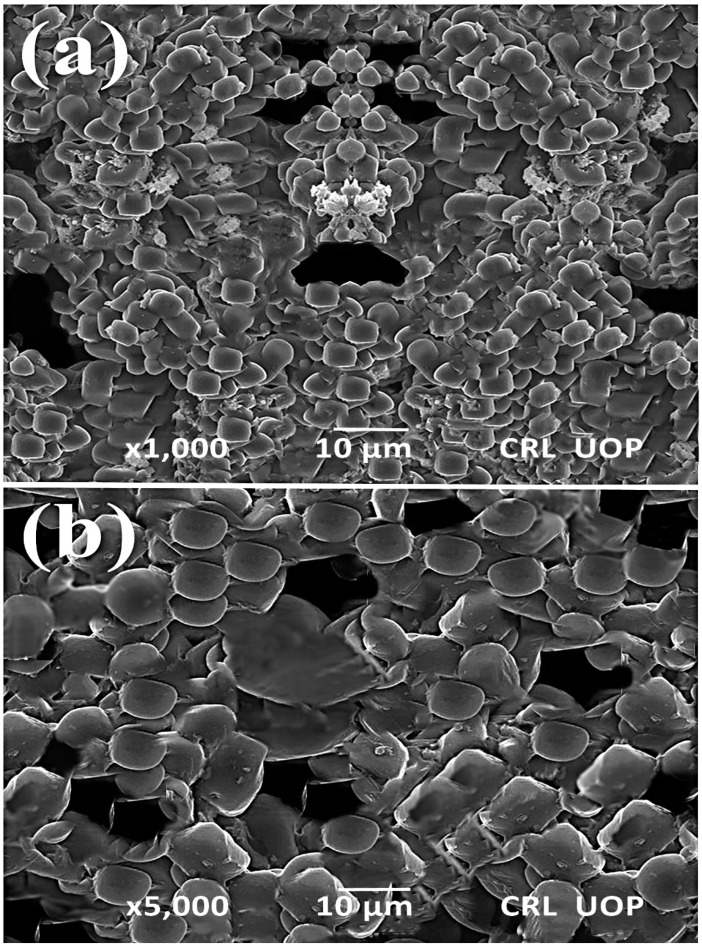
SEM photograph of Titania-based nanocatalyst (**a**) ×1000 (**b**) ×5000.

SEM analysis of TiO_2_ was performed to study the topographies and cross-sections of the nanoparticles [50]. The morphological characteristics of the titanium dioxide nanocatalyst were determined using scanning electron microscopy (SEM). The particle size varied between 68.7 nm and 83.4 nm. This corresponds with the value predicted by the Scherer formula considering the pattern. Our results are correlated with those of Haider et al. [51] and Khalil et al. [52], in which spherically shaped TiO_2_ nanoparticles were synthesized. Figure 4 exhibits an SEM micrograph of the sponge-like form of the titanium dioxide with spherical nanoparticles, and the nanoparticle arrangement was found to be uniform. Larger particles were present due to aggregation of smaller ones.

The energy-dispersive X-ray spectrum (Figure S1) of the titanium dioxide shows that the catalyst contains two elements: titanium and oxygen (Ti = 76% and O = 24%). Similar results were reported by Qamar et al., 2023 [49], and Mukifza et al., 2017 [53], who reported on the purity of titanium dioxide nanoparticles using EDX.

**Figure 5:**
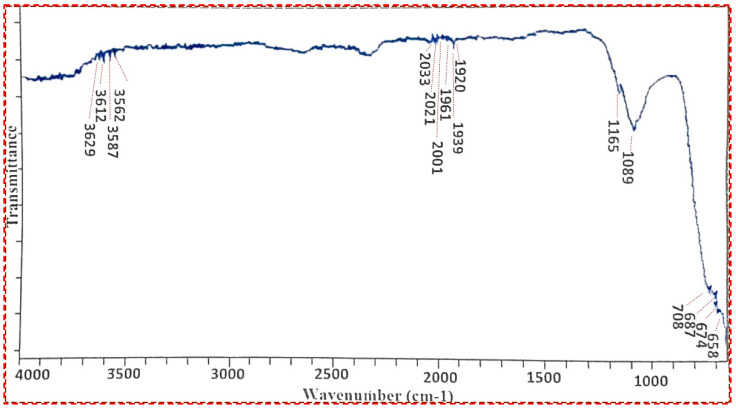
FT-IR spectrum of Titania-based nanocatalyst.

In several fields, such as organic synthesis, polymer science, petrochemical engineering, pharmaceutical manufacture, and food analysis, FT-IR spectroscopy serves as a remarkably advanced tool for process monitoring, compound identification, and mixture component determination [54,55]. In the FT-IR spectra, the weak bands between 3629 cm^−1^ and 1920 cm^−1^ were visible and the weakness in the bands was attributed to the absence of any impurity (Figure 5). However, the strong bands at 658 cm^−1^ and 708 cm^−1^ clearly indicate the presence of Ti-O-Ti vibrations during the stretching of titanium dioxide nanocrystals. Our results are in close accordance with those of Thakur et al., 2019 [56].

### 3.2. Preliminary Study of Feedstock

An economical and ecofriendly biodiesel was synthesized from *Azadiracta indica* seed oil (AISO). The presence of more than 20% oil content makes a given feedstock suitable for biodiesel production. *Azadiracta indica* seeds have 39% oil content, which is high when compared with the several other nonedible feedstocks, such as *Cucumis melo* var. *Agrestis* [39,57,58], *Bischofia javanica*, *Solanum surratense*, *Diospyros lotus* [59], *Jatropha curcus* [60], etc. The seed oil contains 68.98% unsaturated fatty acids (61.01% oleic acid, 8.97% linoleic acid) and 31.02% saturated fatty acids (15.91% palmitic acid, 15.11% stearic acid). *Azadiracta indica* seed oil was found to have high free fatty acid (FFA) content (6.52 mg/g KOH).

### 3.3. Process Parametric Optimization of Transesterification

In this study, a box Bunken design (BBD) was used to construct an experimental matrix including various independent reaction parameters, such as the methanol–oil molar ratio (3:1 to 15:1), the reaction time (60–360 min), the catalyst concentration (0.5–3.5 wt.%), and the reaction temperature (60–120 °C), to optimize the biodiesel yield; this was found to range from 43% to 95%. Utilizing advanced multiple regression analysis, a polynomial equation that incorporates the coefficients of the comprehensive regression model was derived, and its statistical significance was confirmed. The quadratic polynomial equation (Equation (5)), designed to maximize biodiesel yield (%), is as follows (coded factors: alcohol–oil ratio—A; time of reaction—B; catalyst concentration—C; reaction temperature—D).

Biodiesel Yield (wt.%) = +61.31 + 0.4931 ∗ A + 4.66 ∗ B + 0.7691 ∗ C + 0.83 ∗ D + 4.37 ∗ AB + 0.51 ∗ AC + 5.37 ∗ AD + 8.10 ∗ BC + 0.62 ∗ BD + 1.31 ∗ CD − 7.75 ∗ A^2^ + 7.83 ∗ B^2^ + 1.36 ∗ C^2^ + 3.99 ∗ D^2^ (5)

Table 1 displays comprehensive experimental results of the different transesterification optimization reactions. The ANOVA results show a statistical relationship between the biodiesel yield and the influences of the four reaction variables on the yield. The *p*-value and F-value have been computed to evaluate the significance of the associated coefficient. The model is significant and successfully predicts the biodiesel yield according to the ANOVA results, with a *p*-value of 0.0485. The probability value is used to express the model significance (less or more than 0.05). The quadratic terms A^2^ (Met: Oil) and C^2^ catalyst loading were significant, while the B^2^ reaction time and the D^2^ reaction temperature were insignificant (*p*-value > 0.05). If these quadratic terms were removed, the model quality might be raised. These quadratic terms are included since they relate to the model hierarchy (Table 2). The significance of the model can be shown by the insignificant lack-of-fit value, that shows it is not identical to pure error and is incomparable to it also [61,62].

**Figure 6:**
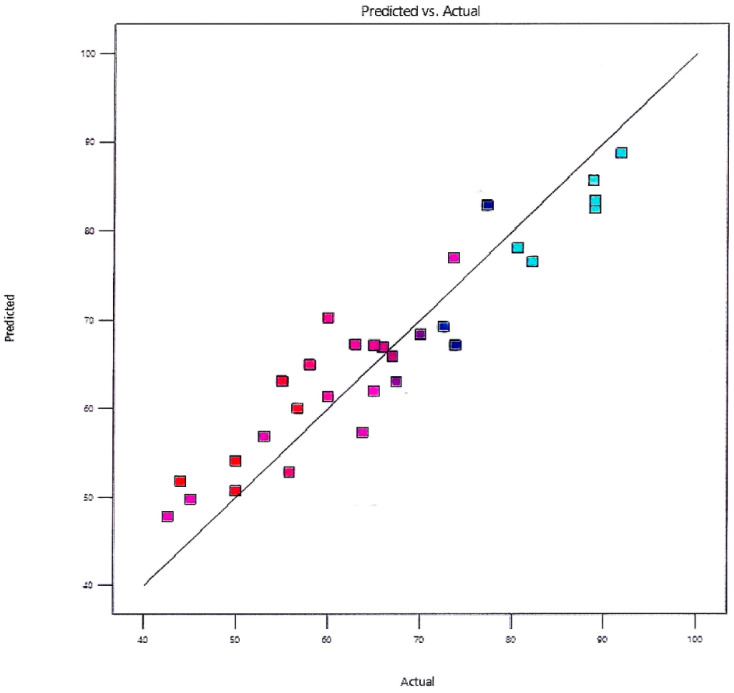
Predicted yield vs. actual yield.

The straight-line graph in Figure 6 shows the high level of agreement between the experimental trial yields and the expected yields. The actual and predicted yields also showed a strong link in the form of a straight line [63].

**Figure 7:**
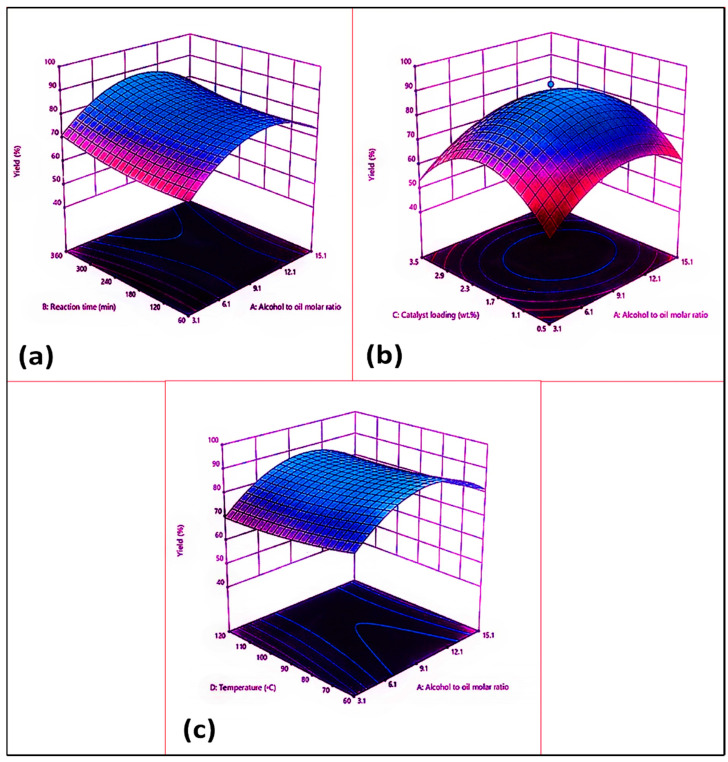
Response surface plots of *Azidhiracta indica* biodiesel: (**a**) alcohol–oil molar ratio and reaction time; (**b**) alcohol–oil molar ratio and catalyst loading (%); (**c**) alcohol–oil molar ratio and reaction temperature.

Figure 7a shows the combined impact of the oil–methanol ratio (A) and the reaction time (B) on the biodiesel yield, while the other factors, such as the catalyst concentration (2%) and the reaction temperature (90 °C) remained constant. The maximum biodiesel yield (95%) was achieved with a 1:9 oil–methanol ratio and a 360 min reaction time. The 3D surface plot clearly shows how raising the oil–methanol molar ratio effectively increases the biodiesel yield.

A suitable oil–methanol ratio must be established within a particular time frame to maximize the biodiesel yield. Therefore, the reversible transesterification reaction needs the extra alcohol to maintain the reaction equilibrium towards a higher conversion of triglycerides into their respective FAMEs. The maximum biodiesel yield (95%) was reached with a 1:9 oil–methanol molar ratio and a 360 min run time (run 19). A reduced methanol–oil molar ratio (3:1) lowers the FAME yield to 67% under similar reaction conditions (run 4). This outcome could be explained by the extent to which the methanol interacts with the oil. The conversion of triglycerides into their corresponding methyl esters was similarly reduced to 51% when the reaction time was further decreased to 60 min (run 13). Transesterification was remarkably influenced by the reaction temperature. A previous study on biodiesel production from waste edible oil (WCO) and corn kernel oil as feedstock indicated that the reaction time has a significant impact on the final product, i.e., the biodiesel yield. Similar behavior was observed in a study by Olutoye et al., in which the maximum FAME yield was achieved during a longer reaction time [64].

The combined impact of the oil–methanol molar ratio (A) and the catalyst loading (C) on the biodiesel yield is depicted in Figure 7b, while other factors, such as reaction time (360 min) and reaction temperature (90 °C), remained constant. The highest biodiesel yield (95%) was attained with 1:9 oil–methanol ratio and a 2 wt.% catalyst loading. The ideal oil–methanol ratio must be established within a given catalyst amount to maximize the biodiesel yield. When the molar ratio was decreased to 1:3 through lowering the catalyst concentration (0.5 wt.%), a sudden fall in biodiesel yield was noticed, i.e., 68% (run 5). Similarly, a drop in biodiesel yield (49%) was noticed when both the molar ratio and the catalyst concentration were increased to the maximum set limits, i.e., 1:15 and 3.5 wt.%, while keeping the other variables at optimal level (run 25). Hence, it was observed that any increase or decrease in either the molar ratio or the catalyst amount resulted in a decline in the FAME yield that could be attributed to the reduced mass transfer and the interaction between the reactant and the catalyst; these led to emulsification, which made the separation process occur. As a result, the biodiesel yield was reduced.

The combined impact of the oil–alcohol molar ratio (A) and the reaction temperature (D) on the biodiesel yield is shown in Figure 7c. The maximum biodiesel yield (95%) was achieved through an increase in temperature of up to 90 °C with a 1:9 oil–methanol molar ratio (Run 19). A decrease in the reaction temperature lowered the yield of the methyl esters in run 16; here, at constant optimum reaction conditions, a decrease in reaction temperature to 60 °C resulted in an 88% biodiesel yield. This is because there was an insufficient activation energy in the reactants to allow them to be converted into their respective products. Inversely, a high temperature beyond the threshold level, i.e., >90 °C (for instance 120 °C), caused the evaporation of alcohol, triggering a reduction in the FAME percentage to 61% (run 20). This is certainly significant because the three-phase thermodynamic transformation requires a sufficient quantity of energy (heat) to overcome the dispersed effect of molecules throughout the catalytic solvent and oily interfaces.

Catarino et al. [65] used calcium diglyceroxide (CaD) as a catalyst in their study to produce biodiesel from nonedible oil via transesterification. They assessed the catalyst’s efficiency, its resistance to air exposure, and the amount of calcium leaching. Calcium oxide (CaO) synthesized from scallop shells recovered from food waste and calcined at 900 °C to make the CaD catalyst. The CaO served as a benchmark catalyst. CaD had lower catalytic activity than CaO under standard reaction conditions, which included a 2.5 h reaction time, methanol reflux at a specific temperature, a 5% catalyst loading based on the amount of oil, and a methanol–oil ratio of 12:1. This led to 92% biodiesel yield with CaO [65]. Puna et al. observed an S-shaped kinetics curve when using CaO as a catalyst and attributed the induction period to the formation of Ca(OH)_2_. The initial slow transesterification phase vanished when Ca(OH)_2_ was used as a catalyst that was synthesized by exposing CaO to water. Additionally, if the catalyst surface was entirely converted into methoxide species, the induction period was longer as compared to the use of CaO alone. This revealed that methoxide species that was strongly bound to calcium is less reactive. On the other hand, when they used calcium diglyceroxide (CaO_diglyc.), prepared from mixing CaO with a mixture of methanol and glycerol, they observed a completely different kinetics curve with no induction period. The faster kinetics and the presence of calcium species in the glycerin phase indicated a major contribution from a homogeneous process. Analyzing the post-reaction catalysts showed the significance of both the surface and the bulk catalyst modifications. Calcium hydroxide appeared as the active phase while calcium diglyceroxide was responsible for catalyst deactivation due to calcium leaching [66].

### 3.4. Biodiesel Characterization

#### 3.4.1. Nuclear Magnetic Resonance (NMR)

**Figure 8:**
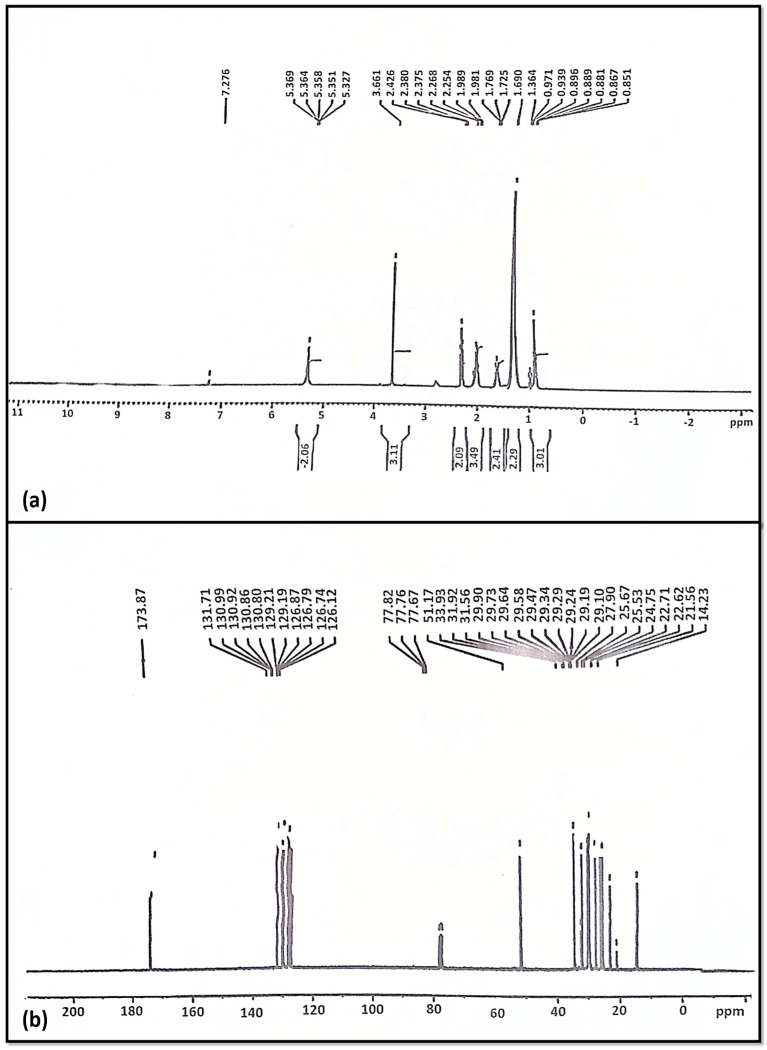
(**a**) ^1^HNMR and (**b**) ^13^CNMR of *Azadhiracta indica* Biodiesel.

The NMR has been found to be an efficient technique for evaluating the quality of biodiesel and the quantification of diesel/biodiesel blends. The proton NMR (^1^HNMR) gives us information about the chemical shifts (the carbon to which the proton is attached), multiplicity (the number of peaks in each group), and the adjacent hydrogen and integrals (the number of hydrogen) [67]. Figure 8 shows the characteristic chemical shifts in the proton NMR (^1^H NMR) spectrum for the produced biodiesel [24].

^1^HNMR spectroscopy is considered to be a simple and fast analytical technique; it yields information on all components of a mixture in one spectrum, usually without derivatization or destruction of the sample. The actual existence of a methoxy proton (–OCH3) was established by the distinctive singlet signal at 3.661 ppm [68]. Our results coincide with previous research, where 3.6 ppm singlet has been reported to be a characteristic peak in ^1^HNMR biodiesel [69,70]. Furthermore, peaks ranging from 2.254 to 2.426 ppm of α-methylene proton (–CH_2_) have been determined in triplets. Various peaks in range of 0.851–0.971 ppm represent terminal methyl protons (CH_3_). The peaks for β-carbonyl methylene protons, ranging from 1.364 to 1.989 ppm, were a major indicator (Figure 8a). Signals of 5.327 ppm and 5.369 ppm supported the presence of olefinic hydrogen [71].

In ^13^CNMR, the existence of long-chain ethylene carbons (–CH_2_–) was established in this particular analysis through elevations at 24.75–33.93 ppm. The *Azadiracta indica* biodiesel content of carbonyl carbon (–C=O), as well as unsaturation position, were confirmed by the peaks at 173.87 ppm and 131.71 ppm. The vinylic group (C=H) is indicated by the peak at 129.19 ppm (Figure 8b). The peak values described previously are evidence that the triglycerides have converted into FAMEs [72,73]. The overall conversion of *Azadhiracta indica* seed oil into biodiesel was estimated using the equation to be 93.4%, close to 95%.

#### 3.4.2. Fourier Transform Infrared Spectroscopy (FT-IR)

**Figure 9:**
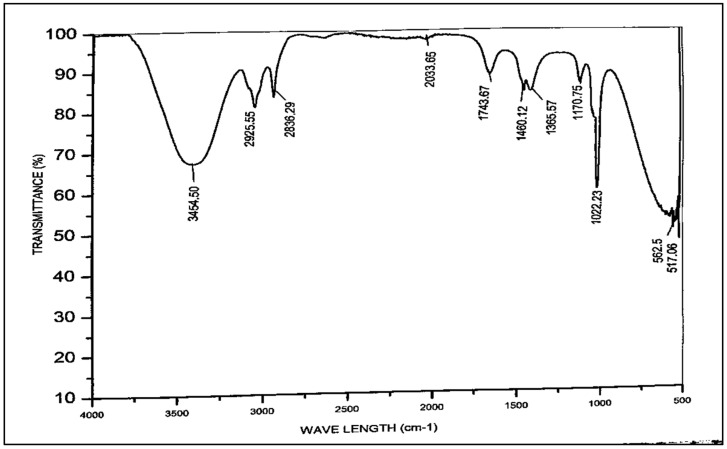
FT-IR of *Azadhiracta indica* biodiesel.

FT-IR spectroscopy was used to identify the functional groups with major bands that correlate to the different bent and stretched modes in the *Azadhiracta indica* biodiesel. The chemical constitution has been examined using FT-IR analysis to validate the methyl ester formation. Distinct functional categories are proven by the relevant peak locations using the FT-IR technique [74]. This specific technique can be used to determine the fat percentage in biodiesel blends. The methyl esters exhibit two primary areas for identification: one represents the bending pattern of the functional group with the carbonyl of the ester at peak areas of 1743.67 cm^−1^ and 1170.75 cm^−1^; alternative band was for C-H, at the peak areas of 2836.29–3454.50 cm^−1^ according to the spectral analysis demonstrated in Figure 9. The subsequent points which emerged at 1743.67 cm^−1^ and 2925.55 cm^−1^ correspond to the ester alkane (C-H) and the carbonyl group (C=O), respectively. Similarly, another peak at 1170.75 cm^−1^ also represents C=O, and the IR band at 572.5 cm^−1^ depicts the C-H rock. The peak, appearing at 1460.12 cm^−1^, shows the C-H bending. The surface of the carbonyl group is sensitive to the unique effects and molecular structure among these peaks [75].

#### 3.4.3. Gas Chromatography–Mass Spectrometry (GC-MS)

The chemical composition of the produced biodiesel from *Azadhiracta indica* seed oil was further analyzed using GC-MS. Eight prominent signals of the FAME have been detected and identified using library software (NO. NIST02) [76]. Figure S2 depicts a sample mass spectrum of the myristic acid methyl ester (C_14_H_28_O_2_) with significant fragment ions (retention period 12.76 min). Other peaks depict the presence of methyl esters of palmitic acid (C_14_H_28_O_2_) (14.88 min), margaric acid (C_16_H_32_O_2_) (15.82 min), linolenic acid (C_17_H_34_O_2_) (16.60 min), stearic acid (C_18_H_36_O_2_) (16.78 min), arachidic acid (C_20_H_40_O_2_) (18.81 min), behenic acid (C_22_H_44_O_2_) (20.08 min), and Heptacosanoic acid (C_27_H_54_O_2_) (21.61 min). Based on the chromatogram, the GC-MS analysis of the *Azadhiracta indica* biodiesel indicates the presence of derivatives, which are chemical compounds derived from seed oil within the fuel. Additionally, the chromatogram reveals that the biodiesel contains methyl palmitate and linoleate as major methyl esters. The analyzer’s graphical user interface showed that the data from the NIST library 02 mass spectrum matched the previously identified components of the biodiesel.

### 3.5. Fuel Properties

The physical–chemical characteristics of the *Azadhiracta indica* biodiesel (AIBD) were quantified and evaluated in comparison to the biodiesel testing standards.

Pure biodiesel was tested for its fuel qualities, including density, cloud point, viscosity, density, sulfur concentration, flash point, and total acid number [67,77]. The results obtained were then compared to both the published research and the standard values set by the European Union (EN 14214) [78], America (ASTM D 6751) [79], and China (GB/T 20828–2007) [34,80]. Table 3 summarizes the data on the outcomes for B-100 on comparison with the standards set by the European Union (EN 14214), American (ASTM D 6751), and China (GB/T 20828-2007).

Fuel density is the mass per unit of volume that may be obtained in the absence of air. The density of the fuel is not itself a significant parameter for the diesel engine, but it is generally related to the fuel energy content. As fuel is sold volumetrically, it can be said that the higher the density, the higher the potential energy on a volume basis will be. It is known that biodiesel has a lower energy content than diesel fuel. However, on a volumetric basis, as the density of biodiesel is higher than diesel, the energy differences become less than before. This AIBD exhibits a density of 0.0832 kg/L according to the ASTM D-1298 analysis [81], compared to high-speed diesel density of 0.897. According to international standards (ASTM, EN), the density of biodiesel must be within the limits of 860–900 kg/m^3^. It is clear in the evidence that AIBD possesses a lower density when compared with that of regular diesel fuel (HSD).

The effects of viscosity can be seen in the quality of atomization, combustion, and engine wear. The high viscosities of biodiesel fuels are reportedly responsible for premature injector fouling, leading to poor atomization. So, the viscosity of biodiesel must satisfy the standards [67]. If a viscous biodiesel is used, then the process of supplying enough fuel to the injection system may lead to failure. However, if the viscosity value of a biodiesel is lower than these limits, then power loss will be an unavoidable result due to leakage. More viscous fuel deposits cause a decrease in combustion rate [82]. Viscosity was lowered due to the ester formation process. Oil with lower viscosity is simpler for pouring and produces finer droplet forms of fuel. The kinematic viscosity (KV) of biodiesel should be in the range of 1.9 to 6.0 centistokes (cSt) at 40 °C (104 °F). This is a measure of the flow characteristics of fuel. According to ASTM D-445 [83], the KV of AIBD is measured at 5.32 at 40 °C, which is within the permissible limits for ASTM requirements, i.e., 1.9–6.0. According to Ameen et al. [6], nonedible seed oil biodiesel has a kinematic viscosity of 4.5 at 40 °C, and is higher than HSD viscosity; this has been reported to be 4.223 [84]. The kinematic viscosity of the AIBD is lower than that of the previously reported biodiesel from *Pongamia pinnata* (5.44 mm^2^/s) and *Monotheca buxifolia* (5.35 mm^2^/s).

The lowest temperature, corrected to a barometric pressure, at which the introduction of an ignition source allows a specimen’s vapors to ignite under test conditions is known as the flash point. This test measures a portion of the residual alcohol in the B100. Fuel flammability can be used to determine storage safety. When all residual alcohol is eliminated, the flash point values of biodiesel fuel are often greater than those of diesel fuel. High flash point fuels are safe to handle, transport, and store. Compared to many other liquid fuels, biodiesel usually has a flash point above 130 °C (266 °F), which makes it comparatively safe in terms of fire risk [85]. The flash point of *Azadhiracta indica* biodiesel is 90 °C, which lies closer to the ASTM D-93 [86], rendering the fuel less risky. The flash point of high-speed diesel (HSD) is between 47.5 °C and 80 °C.

The cloud point (ASTM D2500 [87]) is the temperature at which a liquid biodiesel fuel begins to form visible wax crystals when cooled. It is an important parameter for cold weather performance. Meanwhile, paraffin starts to crystallize or start separating from the solvent at such cold temperatures. The cloud point of biodiesel should be no higher than specific limits, which are typically around −15 °C (5 °F). Lower cloud points are desirable for cold weather operability. The pour point is the lowest temperature at which a liquid biodiesel fuel flows under specific test conditions. It indicates the lowest temperature at which the fuel can be poured or pumped. Biodiesel should have a pour point below −12 °C (10 °F) for adequate cold weather performance. Lower pour points are preferable to ensure that the fuel remains liquid and can be used in colder climates. The pour point and cloud point of AIBD were compared, respectively, to ASTM D-97 [6,88] and ASTM D-2500 [32]. While its cloud point value is −10 °C, which is better than that of HSD, the pour point value of AIBD is −12 °C, in accordance with the ASTM standards.

Phospholipids present in vegetable oils are primarily responsible for the biodiesel sulfur concentration. For the places with high levels of contamination, fuel with a decreased sulfur amount is preferable. Reduced sulfur fuel has been proposed to be better for both the atmosphere and motor longevity. According to Ameen et al., 2018, <0.05% of sulfated ash is present in wild melon seed oil biodiesel. When compared with the ASTM D-4294 standard [89], the sulfur content of AIBD was discovered to be 0.00047% by weight, which was extremely low. It is claimed that the low sulfur content of biofuel makes it favorable as compared to HSD.

The overall amount of natural fatty acids found in the fuel is referred to as the acid value. For neutralizing 1 g of FAMEs, it is expressed in mg KOH/g. Although biodiesel is not naturally acidic, the presence of FFAs in diesel can make it so. For engines, a high acid value is not ideal. According to Ameen et al., 2022, it causes severe corrosion in the fuel-delivery system and internal engine combustion. The acidic content of AIBD was determined using the ASTM D-974 standard [90] to be 0.34 mg KOH/g.

## 4. Membrane Performance and Contribution to System Efficiency

The use of a membrane reactor for biodiesel synthesis brings further challenges such as membrane fouling and durability, system complexity, and initial capital investment. Membrane reactor usage should be based on a thorough cost–benefit analysis that takes into account the unique requirements and limitations of the biodiesel synthesis process [91]. The specific design of membrane reactor for AIBD and TiO_2_ provides following advantages:

Titanium dioxide/Aluminum oxide membrane: The material used for the membrane is important since titanium dioxide/aluminum oxide membranes are known for their strong chemical stability and selectivity. These materials are suitable for the biodiesel synthesis reaction and can survive in extreme conditions, increasing the reactor durability and longevity [92].

Tubular design: When compared to alternative designs, the tubular configuration of the membrane reactor allows for a higher filtering surface area. This expanded surface area improves mass transfer and separation efficiency, allowing for large oil molecule retention and driving higher biodiesel production rates [93].

Sieve plate integration: Integrating sieve plates at the membrane entrance and exit helps to achieve controlled flow distribution and uniform mass transfer across the membrane. This reduces the possibility of channeling and optimizes reaction efficiency by preventing uneven flow and promoting effective separation.

PVC enclosure: Enclosing the tubular membrane with PVC provides physical protection, maintains membrane integrity, and prevents potential damage during operation. PVC is also chemically robust, which is advantageous given the highly reactive nature of the transesterification reaction [94].

Specific reactor dimensions: The exact reactor dimensions were determined to strike a balance between the necessity for adequate filtration surface area and a manageable system size. These measurements were most likely optimized to accommodate the production size and available area.

Pore size: The selection of a pore size of about 0.043 µm is vital for holding oil molecules within the membrane while enabling smaller molecules such as biodiesel to pass through [95]. This size selection improves membrane selectivity, resulting in improved purity in the final biodiesel product [96].

High biodiesel production: The entire design including membrane material, tubular arrangement, sieve plates, and pore size is tailored towards high-quality biodiesel production. This setup is intended to maximize the effective conversion of feedstock into biodiesel by optimizing mass transfer, separation, and reaction conditions.

This membrane reactor configuration plays a crucial role because it approaches biodiesel synthesis holistically. While preserving the longevity and integrity of the equipment, every choice made and component used contributes to better reaction kinetics, product purity, and overall process efficiency, which enhances the biodiesel yield.

## 5. Conclusions

In this study, membrane technology was used to successfully synthesize biodiesel from the nonedible seed oil of *Azadhiracta indica*. TiO_2_ served as a heterogeneous nanocatalyst to synthesize biodiesel in the membrane reactor. *Azadhiracta indica* is a potential feasible source of feedstock for sustainable, low-cost biodiesel synthesis, owing to its high oil content. The high FFA content of seed oil, i.e., 6.52 mg/g KOH, was decreased to less than 2 through esterification followed by transesterification under optimum conditions of a methanol–oil molar ratio of 9:1, 2 wt.% catalyst loading, 90 °C reaction temperature, and 600 rpm agitation rate; a 95% biodiesel yield was achieved. The characterization of titanium dioxide nanoparticles illustrates their pure nature; the particle sizes have excellent thermal durability. Various advanced techniques such as NMR, FT-IR, and GC-MS revealed the existence of high-quality fatty acid methyl esters in the biodiesel sample. Its physicochemical properties were verified in accordance with all the international standards—ASTM D-6571, China GB/T 20828-2007, and EN-14214. In conclusion, membrane technology can drive a significantly intensified reaction processes; the efficient disposal of both nanocatalyst and alcohol showed better transesterification profitability and enabled sustainable biodiesel production. Hence, membrane reactors have been proven to be highly selective, secure, and environmentally friendly, while ensuring a high biodiesel yield.

## Figures and Tables

**Table 1:** Experimental results of transesterification.

	Factor 1	Factor 2	Factor 3	Factor 4	Response
Run	A: Methanol–Oil Molar Ratio	B: Reaction Time	C: Catalyst Loading	D: Reaction Temperature	Biodiesel Yield
		Minutes	Wt.%	°C	%
1.	3:1	60	0.5	60	61
2.	3:1	360	3.5	120	61
3.	3:1	60	3.5	120	77
4.	3:1	360	2	90	67
5.	3:1	360	0.5	90	68
6.	3:1	60	3.5	60	94
7.	3:1	60	0.5	120	79
8.	3:1	210	2	90	52
9.	3:1	360	3.5	60	43
10.	3:1	360	0.5	120	56
11.	9:1	210	2	60	88
12.	9:1	210	2	120	69
13.	9:1	60	2	90	51
14.	9:1	210	0.5	90	73
15.	9:1	360	0.5	90	66
16.	9:1	360	2	60	88
17.	9:1	210	0.5	60	80
18.	9:1	210	3.5	90	72
19.	9:1	360	2	90	95
20.	9:1	360	2	120	61
21.	15:1	60	2	90	71
22.	15:1	60	0.5	120	68
23.	15:1	60	3.5	60	62
24.	15:1	360	0.5	120	88
25.	15:1	360	3.5	90	49
26.	15:1	360	0.5	60	59
27.	15:1	360	3.5	120	67
28.	15:1	210	3.5	90	45
29.	15:1	210	2	90	55
30.	15:1	60	0.5	60	53

**Table 2:** Analysis of variance (ANOVA) of RSM.

Source	Sum of Squares	df	Mean Square	F-Value	*p*-Value	
Model	5049.46	14	341.03	3.11	0.0085	significant
A—Methanol–oil Molar ratio	304.18	1	4.18	0.0193	0.0914	
B—Reaction Time	128.61	1	379.61	1.75	0.2054	
C—Catalyst loading	98.82	1	10.82	0.0499	0.3262	
D—Reaction Temperature	86.67	1	192.67	0.8894	0.3606	
AB	1.25	1	1.57	0.015	0.0014	
AC	8.00	1	7.57	0.0780	0.780	
AD	13.25	1	14.07	0.1440	0.01	
BC	46.00	1	45.77	0.4721	0.50	
BD	0.0525	1	0.06	0.0007	0.70	
CD	30.01	1	27.8	0.304	0.59	
A^2^	469.87	1	468.53	4.78	0.04	
B^2^	19.44	1	19.00	0.19	0.68	
C^2^	1227.61	1	1226.00	12.40	0.004	
D^2^	8.17	1	8.40	0.08	0.7	
Residual	1472.41	15	99.10			
Lack of Fit	1443.91	11	129.10	14.03	0.5054	not significant
Pure Error	31.50	4	7.8	-	-	
Cor Total	6523.87	29				

**Table 3:** Fuel properties of the *Azadhiracta indica* Biodiesel.

Parameter	ASTM	EN 14214	China GB/T 20828-2007	*Azadhiracta indica* Biodiesel
Density@15 °C, gm/cc	0.8445	-	-	0.897
Kinematic Viscosity@40 °C cSt.	1.9–6.0	3.4–5.0	-	5.32
Flash Point °C	100–170	>120	<130	90
Pour Point °C	−15–16	-	-	−12
Cloud Point °C	−3 to −12	-	-	−10
Sulfur Content, %	0.05	0.020	<0.05	0.00047
Total acid no. mg KOH/g	0.8 max	<0.5	<0.8	0.34

## Data Availability

Data are contained within the article and supplementary materials.

## Supplementary Materials

The following supporting information can be downloaded at: https://www.mdpi.com/article/10.3390/membranes13120889/s1, Figure S1: EDX of Titanium Dioxide; Figure S2: GCMS of *Azadhiracta indica* Biodiesel.

## Abbreviations

AISO	*Azadirachta indica* seed oil
TiO_2_	titanium dioxide/Titania
NPs	nanoparticles
FFA	free fatty acid (ffa) content
XRD	X-ray diffraction
SEM	scanning electron microscopy
EDX	energy-dispersive X-ray spectroscopy
FTIR	Fourier transform infrared spectroscopy
GC-MS	gas chromatography–mass spectrometry
NMR	nuclear magnetic resonance
AIBD	*Azadirachta indica* biodiesel
Oil: Met	oil–methanol molar ratio
FAME	fatty acids methyl esters
RSM	response surface methodology
BBD	box Bunken design
ANOVA	analysis of variance
ASTM	American society of testing materials

## References

[1] Amin M.F., Gnida P., Kotowicz S., Małecki J.G., Siwy M., Nitschke P., Schab-Balcerzak E. (2022). Spectroscopic and Physicochemical Investigations of Azomethines with Triphenylamine Core towards Optoelectronics. Materials.

[2] Gnida P., Amin M.F., Pająk A.K., Jarząbek B. (2022). Polymers in High-Efficiency Solar Cells: The Latest Reports. Polymers.

[3] Moyo L., Iyuke S., Muvhiiwa R., Simate G., Hlabangana N. (2020). Application of response surface methodology for optimization of biodiesel production parameters from waste cooking oil using a membrane reactor. S. Afr. J. Chem. Eng..

[4] Guo M., Song W., Buhain J. (2015). Bioenergy and biofuels: History, status, and perspective. Renew. Sustain. Energy Rev..

[5] Habte L., Shiferaw N., Mulatu D., Thenepalli T., Chilakala R., Ahn J.W. (2019). Synthesis of nano-calcium oxide from waste eggshell by sol-gel method. Sustainability.

[6] Ameen M., Zafar M., Ramadan M.F., Ahmad M., Makhkamov T., Bokhari A., Mubashir M., Chuah L.F., Show P.L. (2023). Conversion of novel non-edible *Bischofia javanica* seed oil into methyl ester via recyclable zirconia-based phyto-nanocatalyst: A circular bioeconomy approach for eco-sustenance. Environ. Technol. Innov..

[7] Shuit S.H., Tan S.H. (2019). Esterification of palm fatty acid distillate with methanol via single-step pervaporation membrane reactor: A novel biodiesel production method. Energy Convers. Manag..

[8] Ameen M., Ahmad M., Zafar M., Munir M., Abbas M.M., Sultana S., Elkhatib S.E., Soudagar M.E.M., Kalam M. (2022). Prospects of catalysis for process sustainability of eco-green biodiesel synthesis via transesterification: A state-of-the-art review. Sustainability.

[9] Kurre S.K., Yadav J., Khan M.K., Yadav P., Dhebar M.M., Rawat B.S. (2023). Experimental evaluation of performance and emission of diesel engine fueled with nano-material titanium dioxide (TiO_2_) nanoparticles supplemented diesel-biodiesel-ethanol blends. Mater. Today Proc..

[10] Gardy J., Hassanpour A., Lai X., Ahmed M.H. (2016). Synthesis of Ti (SO_4_) O solid acid nano-catalyst and its application for biodiesel production from used cooking oil. Appl. Catal. A Gen..

[11] Oprescu E.E., Velea S., Doncea S., Radu A., Stepan E., Bolocan I. (2015). Biodiesel from algae oil with high free fatty acid over amphiphilic solid acid catalyst. Chem. Eng. Trans..

[12] Anuradha S., Raj K., Vijayaraghavan V., Viswanathan B. (2014). Sulphated Fe_2_O_3_-TiO_2_ catalysed transesterification of soybean oil to biodiesel. Indian J. Chem. Sect. A.

[13] Emeji I.C., Afolabi A.S., Abdulkareem A.S., Kalala J. Characterization and kinetics of biofuel produced from waste cooking oil. Proceedings of the World Congress on Engineering and Computer Science.

[14] Wen Z., Yu X., Tu S.-T., Yan J., Dahlquist E. (2010). Biodiesel production from waste cooking oil catalyzed by TiO_2_–MgO mixed oxides. Bioresour. Technol..

[15] Kawashima A., Matsubara K., Honda K. (2008). Development of heterogeneous base catalysts for biodiesel production. Bioresour. Technol..

[16] Baroutian S., Aroua M.K., Raman A.A.A., Sulaiman N.M. (2011). A packed bed membrane reactor for production of biodiesel using activated carbon supported catalyst. Bioresour. Technol..

[17] Saidi M., Moradi P. (2020). Conversion of biodiesel synthesis waste to hydrogen in membrane reactor: Theoretical study of glycerol steam reforming. Int. J. Hydrogen Energy.

[18] Sokač T., Gojun M., Tušek A.J., Šalić A., Zelić B. (2020). Purification of biodiesel produced by lipase catalysed transesterification by ultrafiltration: Selection of membranes and analysis of membrane blocking mechanisms. Renew. Energy.

[19] Goswami K.P., Pugazhenthi G. (2021). Effect of binder concentration on properties of low-cost fly ash-based tubular ceramic membrane and its application in separation of glycerol from biodiesel. J. Clean. Prod..

[20] Olagunju O.A., Musonge P., Kiambi S.L. (2022). Production and Optimization of Biodiesel in a Membrane Reactor, Using a Solid Base Catalyst. Membranes.

[21] Tajziehchi K., Sadrameli S. (2020). Optimization for free glycerol, diglyceride, and triglyceride reduction in biodiesel using ultrafiltration polymeric membrane: Effect of process parameters. Process. Saf. Environ. Prot..

[22] Mahboubi A., Ylitervo P., Doyen W., De Wever H., Taherzadeh M.J. (2016). Reverse membrane bioreactor: Introduction to a new technology for biofuel production. Biotechnol. Adv..

[23] Ko M.J., Park H.J., Hong S.Y., Yoo Y.J. (2012). Continuous biodiesel production using in situ glycerol separation by membrane bioreactor system. Bioprocess Biosyst. Eng..

[24] Ameen M., Ahmad M., Zafar M., Sultana S. (2021). Oil Extraction Techniques for Bioenergy Production: A Systematic Review. J. Biomat. Bioprod. Tech..

[25] Gao L., Xu W., Xiao G. (2017). Modeling of biodiesel production in a membrane reactor using solid alkali catalyst. Chem. Eng. Process. Process Intensif..

[26] Hayyan M., Mjalli F.S., Hashim M.A., AlNashef I.M. (2010). A novel technique for separating glycerine from palm oil-based biodiesel using ionic liquids. Fuel Process. Technol..

[27] Anjum F., Mir A., Shakir Y., Zafar M., Sultana S., Ameen M., Ahmad M. (2022). Seed Coat Morphology and Sculpturing of Selected Invasive Alien Plants from Lesser Himalaya Pakistan and Their Systematic Implications. BioMed Res. Int..

[28] Jabeen S., Zafar M., Ahmad M., Althobaiti A., Ozdemir F., Kutlu M., Makhkamov T., Sultana S., Ameen M., Majeed S. (2023). Ultra-sculpturing of seed morphotypes in selected species of genus *Salvia* L. and their taxonomic significance. Plant Biol..

[29] Dutta R., Sarkar U. (2023). Design modifications and scale-up of a novel soxhlet apparatus: Optimization of batch extraction of a biofuel oil from Crotalaria juncea seeds. Sustain. Energy Technol. Assess..

[30] Idibie C., Awatefe K., Ogboru R. (2020). Biodiesel production from dika seed (*Irvingia gabonensis*) oil via soxhlet extraction and transesterification reaction. J. Chem. Soc. Niger..

[31] Gad M., El-Shafay A., Abu Hashish H. (2020). Assessment of diesel engine performance, emissions and combustion characteristics burning biodiesel blends from jatropha seeds. Process. Saf. Environ. Prot..

[32] Ameen M., Zafar M., Nizami A.-S., Ahmad M., Munir M., Sultana S., Usma A., Rehan M. (2022). Biodiesel Synthesis from *Cucumis melo* var. agrestis Seed Oil: Towards Non-Food Biomass Biorefineries. Front. Energy Res..

[33] Jan H.A., Saqib N.U., Khusro A., Sahibzada M.U.K., Rauf M., Alghamdi S., Almehmadi M., Khandaker M.U., Emran T.B., Mohafez H. (2022). Synthesis of biodiesel from *Carthamus tinctorius* L. oil using TiO_2_ nanoparticles as a catalyst. J. King Saud Univ.-Sci..

[34] Bahal M., Kaur N., Sharotri N., Sud D. (2019). Investigations on Amphoteric Chitosan/TiO2 Bionanocomposites for Application in Visible Light Induced Photocatalytic Degradation. Adv. Polym. Technol..

[35] Al-hakimi A.N., Alminderej F., Alhagri I.A., Al-Hazmy S.M., Farea M., Abdallah E. (2023). Inorganic nanofillers TiO_2_ nanoparticles reinforced host polymer polypyrrole for microelectronic devices and high-density energy storage systems. J. Mater. Sci. Mater. Electron..

[36] Khalil R., Kelany N.A., Ibrahim M.A., Al-Senani G.M., Mostafa A.M. (2023). Linear and Nonlinear Optical Properties of PVA: SA Blend Reinforced by TiO_2_ Nanoparticles Prepared by Flower Extract of Aloe Vera for Optoelectronic Applications. Coatings.

[37] Leela S. (2023). Structural and Optical Property of Fe Doped TiO_2_ Anatase Nanoparticle by Sol Gel Route Synthesis. SSRN.

[38] Baroutian S., Aroua M., Abdul Aziz A., Sulaiman N.M. (2012). TiO_2_/Al_2_O_3_ membrane reactor equipped with a methanol recovery unit to produce palm oil biodiesel. Int. J. Energy Res..

[39] Ameen M., Zafar M., Ahmad M., Shaheen A., Yaseen G. (2018). Wild melon: A novel non-edible feedstock for bioenergy. Pet. Sci..

[40] Ahmad M., Khan A.M., Abbas Q., Arfan M., Mahmood T., Zafar M., Raza J., Sultana S., Akhtar M.T., Ameen M. (2022). Implication of scanning electron microscopy as a tool for identification of novel, nonedible oil seeds for biodiesel production. Microsc. Res. Tech..

[41] Deviren H., Aydın H. (2023). Production and physicochemical properties of safflower seed oil extracted using different methods and its conversion to biodiesel. Fuel.

[42] Singh D., Sharma D., Soni S., Sharma S., Sharma P.K., Jhalani A. (2019). A review on feedstocks, production processes, and yield for different generations of biodiesel. Fuel.

[43] Wan Osman W.N.A., Badrol N.A.I., Samsuri S. (2023). Biodiesel Purification by Solvent-Aided Crystallization Using 2-Methyltetrahydrofuran. Molecules.

[44] Sutapa I.W., Sarti, Putnarubun C., Kamari A.B., Bandjar A. (2023). Biodiesel production using *Calophyllum inophyllum* L. oil and CaO as catalyst in the microwave assisted reactor. AIP Conf. Proc..

[45] Farbod M., Khademalrasool M. (2011). Synthesis of TiO_2_ nanoparticles by a combined sol–gel ball milling method and investigation of nanoparticle size effect on their photocatalytic activities. Powder Technol..

[46] Salari M., Marashi P., Rezaee M. (2009). Synthesis of TiO_2_ nanoparticles via a novel mechanochemical method. J. Alloys Compd..

[47] Noor C.W.M., Fhatihah A.N., Mamat R., Norsani W.M., Nurdiyana W., Khasbi M.N. (2023). Properties Study of B20 Palm-Methyl Ester Biodiesel Added with Oxide Nanoparticle towards Green Marine Fuels. J. Adv. Res. Appl. Sci. Eng. Technol..

[48] Bharti A., Debbarma S., Das B. (2023). Effect of hydrogen enrichment and TiO_2_ nanoparticles on waste cooking palm biodiesel run CRDI engine. Int. J. Hydrogen Energy.

[49] Qamar O.A., Jamil F., Hussain M., Bae S., Inayat A., Shah N.S., Waris A., Akhter P., Kwon E.E., Park Y.-K. (2023). Advances in synthesis of TiO_2_ nanoparticles and their application to biodiesel production: A review. Chem. Eng. J..

[50] Chijioke-Okere M.O., Hir Z.A.M., Ogukwe C.E., Njoku P.C., Abdullah A.H., Oguzie E.E. (2021). TiO2/Polyethersulphone films for photocatalytic degradation of acetaminophen in aqueous solution. J. Mol. Liq..

[51] Haider A.J., Anbari R.H.A., Kadhim G.R., Salame C.T. (2017). Exploring potential Environmental applications of TiO2 Nanoparticles. Energy Procedia.

[52] Khalil N.F., Ridha A.M., Al-Mashhadani M.K. Modification of carbon cloth/polyaniline nano-titanium dioxide composite electrodes for enhancement microbial fuel cells (MFCs). Proceedings of the AIP Conference Proceedings.

[53] Ahmad Mukifza H., Awang H., Yusof S., Farid E. (2017). Experimental analysis of titanium dioxide synthesis from synthetic rutile waste using a moderate acid concentration and temperature. Acta Phys. Pol. A.

[54] Paradisi E., Plaza-González P.J., Baldi G., Catalá-Civera J.M., Leonelli C. (2023). On the use of microwaves during combustion/calcination of N-doped TiO2 precursor: An EMW absorption study combined with TGA-DSC-FTIR results. Mater. Lett..

[55] Dineshkumar V., Pryanka J., Ramasamy V., Rajesh R., Selvam C.P. Analysis of green synthesis and characterization of TiO_2_ nanoparticle from lemon peel extract. Proceedings of the AIP Conference Proceedings.

[56] Thakur B., Kumar A., Kumar D. (2019). Green synthesis of titanium dioxide nanoparticles using *Azadirachta indica* leaf extract and evaluation of their antibacterial activity. S. Afr. J. Bot..

[57] Narayan J. (2015). Organogenesis in *Cucumis melo* L. var. *agrestis* Naudin. Rie Consult. Meet-Cum-Semin. Sci. Educ..

[58] Jorge N., da Silva A.C., Malacrida C.R. (2015). Physicochemical characterisation and radical-scavenging activity of Cucurbitaceae seed oils. Nat. Prod. Res..

[59] Ameen M., Zafar M., Ahmad M., Ramadan M.F., Eid H.F., Makhkamov T., Yuldashev A., Mamarakhimov O., Nizomova M., Isaifan R.J. (2023). Assessing the Bioenergy Potential of Novel Non-Edible Biomass Resources via Ultrastructural Analysis of Seed Sculpturing Using Microscopic Imaging Visualization. Agronomy.

[60] Basumatary B., Brahma S., Nath B., Basumatary S.F., Das B., Basumatary S. (2023). Post-harvest waste to value-added materials: *Musa champa* plant as renewable and highly effective base catalyst for *Jatropha curcas* oil-based biodiesel production. Bioresour. Technol. Rep..

[61] Simsek S., Uslu S., Simsek H. (2022). Proportional impact prediction model of animal waste fat-derived biodiesel by ANN and RSM technique for diesel engine. Energy.

[62] Pali H.S., Sharma A., Kumar N., Singh Y. (2021). Biodiesel yield and properties optimization from Kusum oil by RSM. Fuel.

[63] Karimi S., Saidi M. (2022). Biodiesel production from Azadirachta India-derived oil by electrolysis technique: Process optimization using response surface methodology (RSM). Fuel Process. Technol..

[64] Olutoye M., Wong S., Chin L., Amani H., Asif M., Hameed B. (2016). Synthesis of fatty acid methyl esters via the transesterification of waste cooking oil by methanol with a barium-modified montmorillonite K10 catalyst. Renew. Energy.

[65] Catarino M., Martins S., Dias A.P.S., Pereira M.F.C., Gomes J. (2019). Calcium diglyceroxide as a catalyst for biodiesel production. J. Environ. Chem. Eng..

[66] Dias A.P.S., Puna J., Gomes J., Correia M.J.N., Bordado J. (2016). Biodiesel production over lime. Catalytic contributions of bulk phases and surface Ca species formed during reaction. Renew. Energy.

[67] Jan H.A., Osman A.I., Al-Fatesh A.S., Almutairi G., Surina I., Al-Otaibi R.L., Al-Zaqri N., Kumar R., Rooney D.W. (2023). Biodiesel production from *Sisymbrium irio* as a potential novel biomass waste feedstock using homemade titania catalyst. Sci. Rep..

[68] Argaw Shiferaw K., Mathews J.M., Yu E., Choi E.-Y., Tarte N.H. (2023). Sodium Methoxide/Zeolite-Supported Catalyst for Transesterification of Soybean Waste Cooking Oil for Biodiesel Production. Inorganics.

[69] Hasnain S.M.M., Chatterjee R., Ranjan P., Kumar G., Sharma S., Kumar A., Salah B., Ullah S.S. (2023). Performance, Emission, and Spectroscopic Analysis of Diesel Engine Fuelled with Ternary Biofuel Blends. Sustainability.

[70] Yatish K., Omkaresh B., Kattimani V.R., Lalithamba H., Sakar M., Balakrishna R.G. (2023). Solar energy-assisted reactor for the sustainable biodiesel production from *Butea monosperma* oil: Optimization, kinetic, thermodynamic and assessment studies. Energy.

[71] Daimary N., Eldiehy K.S., Bora N., Boruah P., Rather M.A., Mandal M., Bora U., Deka D. (2023). Towards integrated sustainable biofuel and chemical production: An application of banana pseudostem ash in the production of biodiesel and recovery of lignin from bamboo leaves. Chemosphere.

[72] Liew C.S., Mong G.R., Lim J.W., Raksasat R., Rawindran H., Hassan M.A., Lam M.K., Khoo K.S., Zango Z.U. (2023). Low-temperature thermal pre-treated sewage sludge for feeding of black soldier fly (*Hermetia illucens*) larvae: Protein, lipid and biodiesel profile and characterization. Renew. Sustain. Energy Rev..

[73] Mamedov I., Javadova O., Iskakov R. (2023). Testing of n-butanol and eucalyptus essential oil as additivities of cottonseed biodiesel-diesel blends. Indian J. Chem. Technol..

[74] Zeeshan M., Ghazanfar S., Tariq M., Asif H.M., Hussain A., Usman M., Khan M.A., Mahmood K., Sirajuddin M., Imran M. (2023). Synthesis of novel ternary NiO–CdO-Nd_2_O_3_ nanocomposite for biodiesel production. Renew. Energy.

[75] Joshi N.C., Gururani P., Bhatnagar P., Kumar V., Vlaskin M.S. (2023). Advances in Metal Oxide-based Nanocatalysts for Biodiesel Production: A Review. ChemBioEng Rev..

[76] Kumar S., Manasa V., Madhubalaji C., Tumaney A., Giridhar P. (2023). GC/MS quantification of individual fatty acids of selected green leafy vegetable foliage and their biodiesel attributes. Grasas Y Aceites.

[77] Jamaluddin N., Riayatsyah T.M.I., Silitonga A.S., Mofijur M., Shamsuddin A.H., Ong H.C., Mahlia T.M.I., Rahman S.A. (2019). Techno-Economic Analysis and Physicochemical Properties of *Ceiba pentandra* as Second-Generation Biodiesel Based on ASTM D6751 and EN 14214. Processes.

[78] https://www.en-standard.eu/une-en-14214-2013-v2-a2-2019-liquid-petroleum-products-fatty-acid-methyl-esters-fame-for-use-in-diesel-engines-and-heating-applications-requirements-and-test-methods/.

[79] https://www.astm.org/d6751-20a.html.

[80] https://www.chinesestandard.net/PDF/English.aspx/GBT20828-2007.

[81] https://www.astm.org/d1298-12br17e01.html.

[82] Suzihaque M., Syazwina N., Alwi H., Ibrahim U.K., Abdullah S., Haron N. (2022). A sustainability study of the processing of kitchen waste as a potential source of biofuel: Biodiesel production from waste cooking oil (WCO). Mater. Today Proc..

[83] https://www.astm.org/standards/d445.

[84] Aziz A., Ahmad M., Ullah R., Bari A., Khan M.Y., Zafar M., Sultana S., Rozina, Ameen M., Anar M. (2022). Microscopic techniques for characterization and authentication of oil-yielding seeds. Microsc. Res. Tech..

[85] Ávila Vázquez V., Díaz Estrada R.A., Aguilera Flores M.M., Escamilla Alvarado C., Correa Aguado H.C. (2020). Transesterification of non-edible castor oil (*Ricinus communis* L.) from Mexico for biodiesel production: A physicochemical characterization. Biofuels.

[86] https://webstore.ansi.org/standards/astm/astmd9316?gad_source=1&gclid=EAIaIQobChMIwNTh_ZzmggMVQQUGAB0tnQlIEAAYASAAEgLH4vD_BwE|.

[87] https://www.astm.org/d2500-17a.html.

[88] https://ayalytical.com/methods/astm-d97/.

[89] https://www.astm.org/d4294-21.html.

[90] https://www.astm.org/d0974-22.html.

[91] Baral S.S., Dionisi D., Maarisetty D., Gandhi A., Kothari A., Gupta G., Jain P. (2020). Biofuel production potential from wastewater in India by integrating anaerobic membrane reactor with algal photobioreactor. Biomass Bioenergy.

[92] Tayel A., Ramadan A.R., El Seoud O.A. (2018). Titanium dioxide/graphene and titanium dioxide/graphene oxide nanocomposites: Synthesis, characterization and photocatalytic applications for water decontamination. Catalysts.

[93] Khademi M.H., Alipour-Dehkordi A., Nalchifard F. (2023). Sustainable hydrogen and syngas production from waste valorization of biodiesel synthesis by-product: Green chemistry approach. Renew. Sustain. Energy Rev..

[94] Wiley P.E. (2013). Microalgae Cultivation Using Offshore Membrane Enclosures for Growing Algae (OMEGA).

[95] Ameen M., Zafar M., Ahmad M., Sultana S., Makhkamov T., Yuldashev A., Mamarakhimov O., Nasirov M., Kilic O., Ozdemir F.A. (2023). Prospects of Bioenergy Development in Future In Biomass Energy. Reference Module in Earth Systems and Environmental Sciences.

[96] Garg S., Behera S., Ruiz H.A., Kumar S. (2023). A Review on Opportunities and Limitations of Membrane Bioreactor Configuration in Biofuel Production. Appl. Biochem. Biotechnol..

