# Supplementary figures and images for: Cleaner Biofuel Production via Process Parametric Optimization of Nonedible Feedstock in a Membrane Reactor Using a Titania-Based Heterogeneous Nanocatalyst: An Aid to Sustainable Energy Development
Source: Membranes (Basel). 2023 Nov 27;13(12):889. doi: 10.3390/membranes13120889 (PMC10744951; doi:10.3390/membranes13120889)

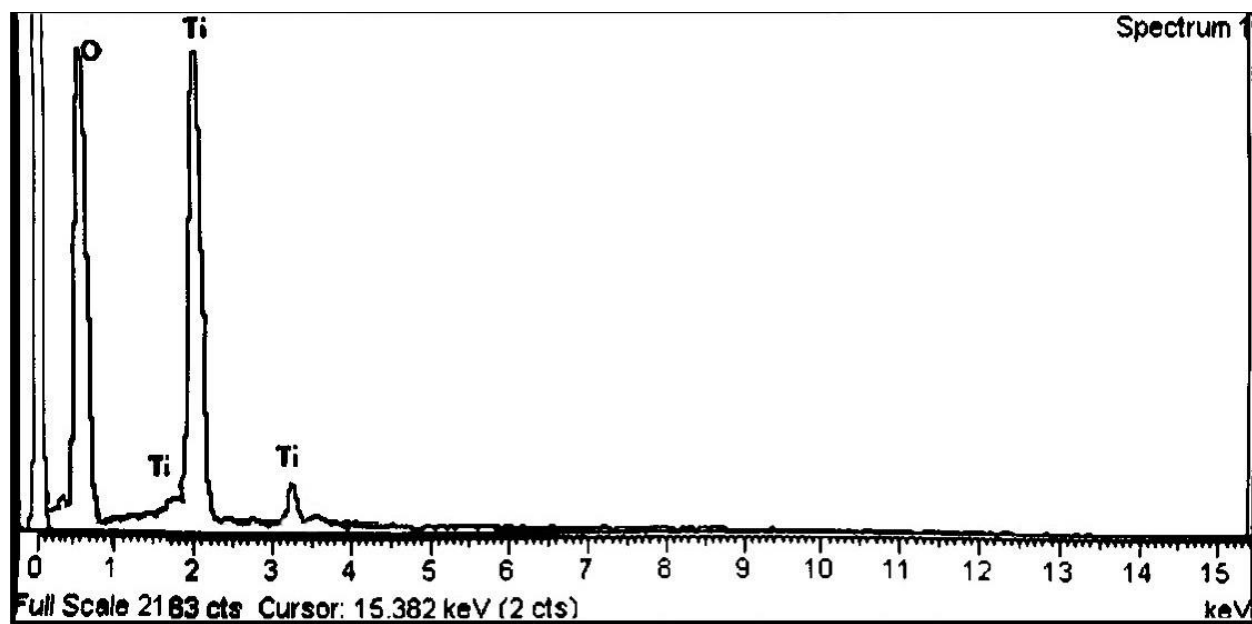

Figure S1 EDX of Titanium Dioxide

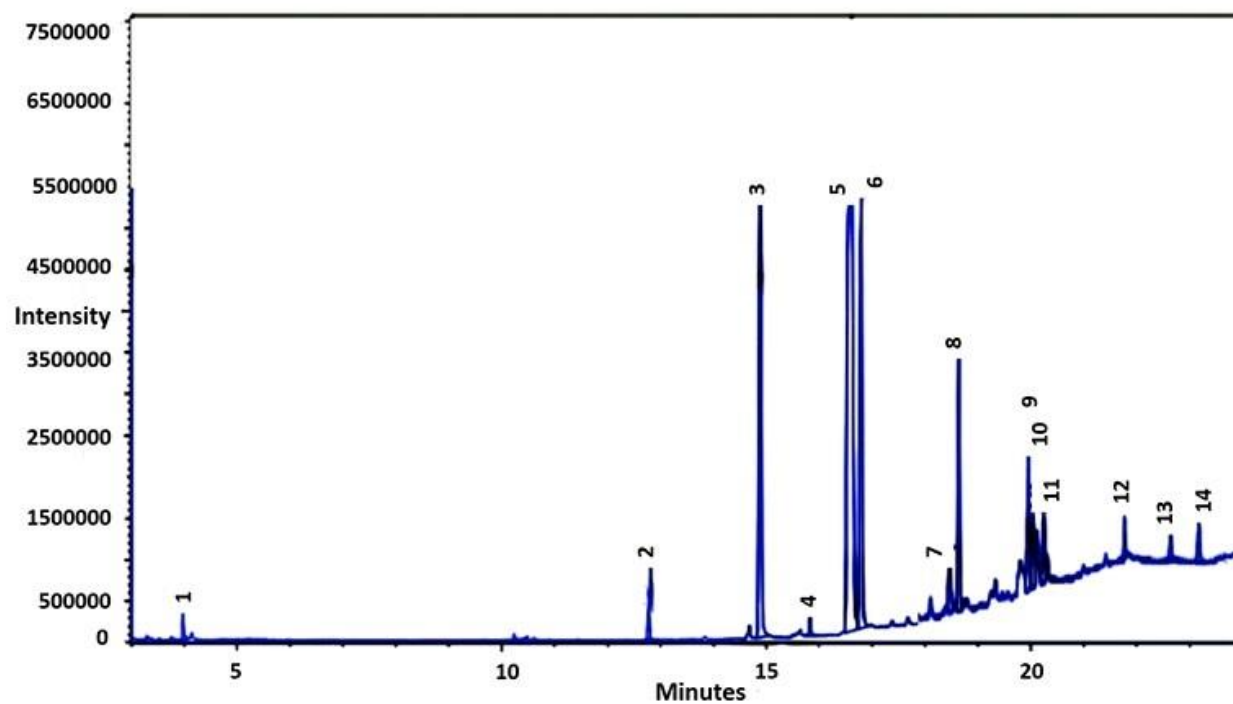

Figure S2 GCMS of *Azadiracta indica* Biodiesel

Supplement: Supplementary file 1 [file membranes-13-00889-s001.zip › membranes-2602985-supplementary.pdf]
